# Novel Biomarkers for Alzheimer's Disease: Plasma Neurofilament Light and Cerebrospinal Fluid

**DOI:** 10.1155/2024/6668159

**Published:** 2024-05-15

**Authors:** Daniel Naawenkangua Abukuri

**Affiliations:** Department of Psychology, University of Ghana, College of Humanity, Accra, Ghana

## Abstract

Neurodegenerative disorders such as Alzheimer's disease (AD) represent an increasingly significant public health concern. As clinical diagnosis faces challenges, biomarkers are becoming increasingly important in research, trials, and patient assessments. While biomarkers like amyloid-*β* peptide, tau proteins, CSF levels (A*β*, tau, and p-tau), and neuroimaging techniques are commonly used in AD diagnosis, they are often limited and invasive in monitoring and diagnosis. For this reason, blood-based biomarkers are the optimal choice for detecting neurodegeneration in brain diseases due to their noninvasiveness, affordability, reliability, and consistency. This literature review focuses on plasma neurofilament light (NfL) and CSF NfL as blood-based biomarkers used in recent AD diagnosis. The findings revealed that the core CSF biomarkers of neurodegeneration (T-tau, P-tau, and A*β*42), CSF NFL, and plasma T-tau were strongly associated with Alzheimer's disease, and the core biomarkers were strongly associated with mild cognitive impairment due to Alzheimer's disease. Elevated levels of plasma and cerebrospinal fluid NfL were linked to decreased [18F]FDG uptake in corresponding brain areas. In participants with A*β* positivity (A*β*+), NfL correlated with reduced metabolism in regions susceptible to Alzheimer's disease. In addition, CSF NfL levels correlate with brain atrophy and predict cognitive changes, while plasma total tau does not. Plasma P-tau, especially in combination with A*β*42/A*β*40, is promising for symptomatic AD stages. Though not AD-exclusive, blood NfL holds promise for neurodegeneration detection and assessing treatment efficacy. Given the consistent levels of T-tau, P-tau, A*β*42, and NFL in CSF, their incorporation into both clinical practice and research is highly recommended.

## 1. Introduction

Alzheimer's disease is a progressive neurodegenerative disease [1] characterized by the presence of amyloid *β* (A*β*) plaques and neurofibrillary tangles consisting of tau protein in the brain [1, 2]. The buildup of A*β* has been recognized as the primary molecular source of the development and progression of Alzheimer's disease. Amyloid *β* is the major constituent of neuritic plaques in Alzheimer's disease [1, 3]. The tau protein is a microtubule-associated protein that supports the movement of essential substances and nutrients throughout the nerve cell [3]. Neurofibrillary tangles are formed when tau protein is abnormal and microtubule structures break down in Alzheimer's disease [3]. Neurons that have plaques around them die, presumably as a result of an immunological response in the surrounding region [3, 4]. Thus, a microscopic analysis of several different brain areas is needed for the final AD diagnosis [2, 3]. The diagnosis is made based on the topographic distribution, morphology, and density of lesions [4, 5]. Interestingly, there have been several reports of Alzheimer's disease [6, 7]. The prevalence of Alzheimer's disease varies across the world, ranging from 5% to 7% in most countries [8]. In the US, African Americans and Latinos have higher prevalence rates compared to non-Latino Whites [6]. Similarly, in the UK, African-Caribbean AD patients show higher rates than non-Latino Whites [7]. Reports indicate that nonindustrialized countries, such as Nigeria (1.4%) and rural India (1.1%), exhibit lower Alzheimer's disease incidence, although rates are increasing in Africa and Asia [9, 10]. Differences in how Alzheimer's disease is identified and higher mortality rates in some countries might explain these variations [10]. Also, lifestyle habits like diet and exercise could affect these differences that we see [9, 10]. In countries with less industry, people are living longer, which means there are older adults who could be at risk for Alzheimer's disease [9, 11]. In the next few decades, more than half of these older adults will likely be in countries that are not very wealthy, reaching around 71% by 2050 [10]. Alzheimer's disease incidence doubles every 10 years after age 60 [10], with minimal sex differences, but more women are affected due to longer life expectancy [11]. The occurrence of new cases of Alzheimer's disease among various ethnic groups becomes more similar once differences in education or socioeconomic status are considered [12]. However, the incidence rates of Alzheimer's disease show variability among different populations and age groups [7, 10]. Studies in China indicate rates similar to those in the US and Europe, ranging from 5/1000 in ages 65-70 to 60-80/1000 in ages 85 and above [8, 13]. In northern California, African Americans have a higher incidence, while Asian Americans, particularly the Japanese, show lower rates [14]. The Cardiovascular Health Study Cognition Study indicates varying estimates, ranging from 32/1000 person-years at age 75-79 to 96/1000 at age 85, with minimal differences between sexes [11]. Incidence continues to increase after age 90+ [15], and long-term studies demonstrate that only 2% of initially healthy participants remain unaffected after over two decades [16].

These rates are not just mere figures; they suggest the significant impact that Alzheimer's disease has on older adults. Alzheimer's disease has been found as the primary cause of death in this age group [8]. A survey conducted by Medicare found that Alzheimer's disease accounted for 19% of all deaths among people aged 65 and older, ranking second after heart failure [17]. However, traditional mortality statistics tend to underestimate Alzheimer's-related deaths, as advanced stages of the disease increase vulnerability to other diseases or infections [17, 18]. Although Alzheimer's disease itself might not directly cause death, in its advanced stages, it can make people more prone to other conditions that might lead to death [17]. Additionally, Alzheimer's disease is a primary contributor to disability, dependency, and mortality in older populations [19]. While there has been considerable progression in understanding how common Alzheimer's disease is and its effects on older individuals, diagnosing the disease has become more complicated [4, 5]. This emphasizes the need for the development and improvement of new tools for screening and diagnosing Alzheimer's disease [20]. The limitations of current diagnostic techniques, the advantages of early diagnosis, and the challenges in accurately diagnosing Alzheimer's disease underscore the need for better diagnostic tools [21]. Early AD diagnosis is important because it enables planning for the future, making critical decisions about care; accessing essential information, resources, and support; and potentially benefiting from available treatments [22]. Early diagnosis can also reduce the financial and emotional impact of AD, improve the quality of life and care, and potentially result in significant cost savings [23]. Interestingly, the challenge of differentiating Alzheimer's disease from other neurodegenerative conditions stems from the lack of reliable biomarkers, despite the rigorous evaluation of thinking and memory abilities in current diagnostic methods [21]. Due to inconsistencies in biomarkers and the need for more accurate and reliable diagnostic methods [24], it is crucial to advance diagnostic techniques to enhance early detection, provide timely treatments, and ultimately improve interventions for Alzheimer's disease [20, 23]. Several biomarkers have emerged in Alzheimer's disease diagnosis, including structural magnetic resonance imaging for amyloid *β* and amyloid pathology observed on PET scans [25, 26], along with markers indicating neuronal injury and assessments of brain metabolism [26, 27]. Nonetheless, the practical application of these biomarkers faces problems stemming from concerns about their invasiveness, considerable expenses, and the limited accessibility of PET imaging [28].

In recent times, blood-based biomarkers have shown promising potential in addressing several of these challenges. They are capable of differentiating between individuals with Alzheimer's disease and those exhibiting normal cognitive function [24, 27]. Studies also suggest that assessing blood metabolites offers insights into the pathological changes associated with Alzheimer's disease [29, 30]. Among these metabolites, reduced levels of thiamine have emerged as a potential diagnostic biomarker for AD, demonstrating a sensitivity of 77.4% and specificity of 78.1% when differentiating AD patients from healthy individuals. The use of the HPLC technique enhances the appeal of this approach due to its accessibility and cost-effectiveness [29]. Another approach involves using autoantibodies to differentiate between mild cognitive impairment (MCI) associated with aging or other neurodegenerative diseases and MCI related to early-stage AD [22, 30]. Additionally, this method is aimed at differentiating early-stage AD from its more advanced forms [24, 26]. Studies testing blood samples from patients with low A*β*42 CSF levels—a sign of early AD or predisposition to rapid disease progression—demonstrated accuracy and specificity levels exceeding 90% [29, 30]. Despite the unavailability of extensive data on blood-based AD biomarkers and validated blood sampling methods, the easiness and noninvasiveness of these approaches facilitate further exploration [30]. Recent progress in diagnostic tools includes blood-based biomarkers, specifically plasma neurofilament light (NfL) and cerebrospinal fluid (CSF) markers [26, 27]. The CSF markers include the 42-amino acid form of A*β*, total tau, and phosphorylated tau [29]. These biomarkers show significant promise in monitoring and screening disease progression [25, 27]. Moreover, blood-based biomarkers differentiate individuals with Alzheimer's disease from those with normal cognitive function [31], including those diagnosed with mild cognitive impairment (MCI), and have been associated with cerebrospinal fluid biomarkers [32, 33]. Increased levels of NfL in the bloodstream are indicative of axonal damage and neuronal injury [34], showing a correlation with cognitive decline, even in individuals initially without cognitive impairment [35]. Similarly, increased plasma p-tau181 levels have been observed in individuals diagnosed with AD, predicting disease progression and cognitive decline in those without impairment and those with MCI [33, 36]. Although these biomarkers are primarily used in research settings, there are ongoing attempts to enhance their accessibility for screening in clinical trials and to potentially integrate them into clinical practice [34, 35]. Despite their growing relevance, blood-based biomarkers have not been thoroughly explored [37–39]. Therefore, this paper is aimed at providing a review of these emerging biomarkers (plasma NfL and CSF), highlighting their significance in Alzheimer's disease pathology. The literature review will further explore their associations with neurodegeneration, cognitive decline, and disease progression. It is aimed at evaluating traditional biomarker limitations and highlighting the benefits of plasma NfL and CSF markers as less invasive, offering crucial clinical insights. This review will substantially contribute to understanding Alzheimer's disease, facilitate drug development, address blood-based biomarker trials, and improve clinical practices.

## 2. Evolution and Major Challenges of Biomarkers for Alzheimer's Disease

Many studies have been conducted regarding the historical development of the biomarkers used to diagnose Alzheimer's disease [40–42]. The use of biomarkers in Alzheimer's disease diagnosis has increased throughout time as a result of scientific recommendations and advances in technology [40, 42]. Although clinical techniques have historically dominated the treatment of Alzheimer's disease, biomarkers are becoming increasingly important in both diagnosis and treatment approaches [39, 42]. Earlier studies have shown that Alzheimer's disease typically presents two neuropathological features in patients' brains: senile plaques, composed of amyloid *β* peptides found outside the cells, and neurofibrillary tangles containing hyperphosphorylated tau proteins within the cells [43]. Thus, the National Institute on Aging and the Alzheimer's Association proposed new diagnostic criteria for Alzheimer's disease in 2018, emphasizing the combination of tau and amyloid PET scans and the analysis of amyloid-*β* and tau proteins in CSF with clinical assessments [43, 44]. This will help in the accurate diagnosis of Alzheimer's disease [44]. These criteria support the use of biomarkers to identify tau tangles and amyloid plaques in the brain before the observable onset of cognitive impairment, thereby facilitating early diagnosis and intervention [42]. At present, many laboratories can effortlessly identify clinical symptoms of Alzheimer's disease using biomarkers because of the development of new diagnostic criteria [42, 44].

### 2.1. Plasma A*β* Species and Tau Forms

Amyloid plaques primarily comprise peptides made from the enzymatic cleavage of *β*-amyloid precursor protein (APP) [45]. The transmembrane protein, which is produced by the chromosome 21 gene, is subject to alternative splicing, leading to the production of several variants. The most often occurring form in the brain is APP 695 [43, 45]. Though its specific role is yet unknown, extracellular matrix and cellular interactions are believed to be modified by APP. Studies conducted on animals have focused on the enzymes that are involved in the metabolism of A*β* peptides, specifically neprilysin and insulin-degrading enzymes (IDE) [41, 45]. IDE's significance lies in its role in degrading A*β* peptides [45], establishing a significant association between degenerative conditions like Alzheimer's disease and type 2 diabetes mellitus (DM2) [45, 46]. Therefore, DM2 is one of the identified risk factors for Alzheimer's disease [46]. Increased A*β* concentrations in plasma and cerebrospinal fluid have been linked to higher insulin levels in people [47]. This may be because IDE breaks down insulin more effectively than A*β* peptides. Thus, increased insulin levels may prevent IDE activity away from A*β* breakdown, showing a relationship between insulin fluctuations and IDE function [46, 47]. Multiple studies included have reported decreased levels of A*β* peptides ending at 42 (A*β*42) in CSF among AD patients [48]. Reduced CSF A*β*42 concentrations in AD could be due to sequestration of A*β*42 in plaques, limiting its clearance into the CSF [49]. Nevertheless, individuals with Creutzfeldt-Jakob disease (CJD) lacking amyloid plaques show a reduction in selective A*β*42 in cerebrospinal fluid [50]. This shows that this hypothesis is not always true [50]. Comparably, cases of bacterial meningitis, which do not produce amyloid plaques but may result in long-term memory loss, had lower A*β*42 levels [51]. These differences point to various underlying processes in many pathological conditions influencing CSF levels of A*β*42. A significant number of explanations have been put up to account for the decline in A*β*42 concentrations in AD-related cerebrospinal fluid [49, 51]. According to one conception, there may be fewer neurons releasing A*β* peptides into the brain parenchyma, which might lead to a decrease in the rate of A*β* production [48, 49]. However, this notion contradicts the observed increased A*β*42 load in brain tissue identified through mass spectrometry analyses [48]. Additionally, if this theory was valid, concentrations of other isoforms like A*β*40 and A*β*38 in CSF should also decrease in AD, which has not been observed [51, 52]. In addition, this notion is challenged by lower A*β*42 levels in Down syndrome and familial AD, two disorders marked by genetically driven A*β* overproduction [52]. Furthermore, because AD and MCI-AD patients share a metabolic course with A*β* peptides, there is an associated increase in soluble APP concentrations, which makes it challenging to correlate decreased synthesis to lower A*β*42 concentrations [49, 52]. Increased A*β*42 degradation is a possible explanation for reduced CSF A*β*42 in AD patients [52, 53]. Though both A*β* peptides are processed by similar enzymes, this process should affect them equally, particularly A*β*40 [49, 53]. In breaking down A*β*1-40 and A*β*1-42, for instance, IDE's efficiency is the same [53]. Moreover, increased A*β*42 degradation should prevent A*β*42 deposits from forming in the brain parenchyma, which would impact amyloid plaques [53]. An interesting but unconfirmed hypothesis argues that the aggregation of A42 monomers into soluble oligomers may be the cause of the decreased CSF A*β*42 concentration in AD [52, 54]. This aggregation can make antibody binding sites distant, affecting antibody epitopes used in ligand-based assays [53, 54]. This hypothesis is consistent with higher amounts of A42 in the brain parenchyma of AD patients and the recent finding of A*β*42 oligomerization in AD. Furthermore, this approach explains why AD patients have increased A*β* oligomer concentrations in their CSF [54].

On the other hand, tau proteins, which are members of the microtubule-associated protein family, are found in both neurons and nonneuronal cells and are encoded by the human tau gene (MAPT) on chromosome 17 [48, 53]. They help with microtubule processes like as nucleation, growth, and bundling, which are regulated by phosphorylation and impact tau-microtubule connections [48]. New research suggests that extracellular tau may play a pathogenic role [52, 53]. In AD, the spread of tau pathology in brain tissue correlates with cognitive decline [53]. Similar to A*β*, tau oligomerization has been considered a potential diagnostic and therapeutic target [51, 52]. Total tau protein concentration, regardless of phosphorylation status, is frequently investigated as a nonspecific measure for neuronal loss in neurodegeneration [53]. Total tau concentrations in CSF are higher in individuals with neuropsychiatric disorders characterized by acute neuronal injury, such as Creutzfeldt-Jakob disease and stroke [51, 53]. However, abnormal hyperphosphorylation of tau is an important characteristic of Alzheimer's disease. In Alzheimer's disease patients with low CSF A42, increased CSF tau phosphorylated at amino acid position 181 (p-tau181) concentrations was found [51, 54]. Similarly, except for corticobasal degeneration, increased p-tau181 levels were found in AD patients when compared to other neurodegenerative conditions [52, 54]. Tau phosphorylation at other locations, such as threonine 231 (pTau231) and serine 235, can differentiate Alzheimer's disease from other related disorders, such as frontotemporal lobar degeneration, vascular dementia, and dementia with Lewy bodies. Furthermore, pTau231 tends to be useful in diagnosing MCI cases that develop into AD during follow-up [48, 52, 54].

### 2.2. Cerebrospinal Fluid Biomarkers of *β*-Amyloid Aggregation, Metabolism, and Pathology in Alzheimer's Disease

#### 2.2.1. CSF Biomarkers of Tau Pathology

The potential to use tau protein in cerebrospinal fluid as an Alzheimer's disease biomarker emerged in 1993 through ELISA techniques using a polyclonal antibody [55, 56]. A subsequent study using monoclonal antibodies confirmed the detection of all tau protein isoforms through ELISA [55, 56]. These studies revealed that CSF tau protein levels, particularly in individuals with AD and MCI, provide accurate changes between mild AD and cognitively healthy individuals [41, 56]. When combined with other CSF proteins, these markers become even more predictive [28]. Increase in levels of total tau protein in CSF are primarily seen in patients with neuropsychiatric disorders marked by neuronal damage or loss [57, 58]. Furthermore, lower levels of A*β*42 in the CSF correlate with AD patients having increased levels of phosphorylated tau protein at amino acid position 181 (p-tau181) [59]. Total tau (T-tau) and p-tau181 levels in the CSF can both predict the outcomes of individuals with motor cognitive impairment as well as determine the severity of neuronal degeneration in progressive Alzheimer's disease [53, 60]. In individuals with normal cognitive function, there exists a significant correlation between brain amyloid levels and both CSF tau and the tau/A*β*42 ratio [49].

#### 2.2.2. CSF Total Tau (T-Tau) and Phosphorylated Tau (p-Tau)

CSF amyloid-beta consists of peptides resulting from the breakdown of the *β*-amyloid precursor protein (APP) by enzymes [61]. Recent studies found important C-terminal forms—A*β*1-40 and A*β*1-42—in the CSF, with A*β*1-42 having a high tendency to accumulate into plaques [45, 62]. The decline in CSF A*β*1-42 observed in AD may come from A*β* accumulation in plaques. The increased levels of A*β*1-42 found in amyloid plaques in the brain and CSF of AD patients likely contribute to the clinical symptoms of AD, considering its absence in other tauopathies [63, 64]. Interestingly, a fifty percent decrease in CSF A*β*42 levels has been observed in AD, which is thought to occur because the peptide becomes trapped in A*β* plaques in the brain [61, 65, 66]. However, CSF A*β* levels cannot explain nearly one-third of the changes in amyloid imaging [67]. In Alzheimer's disease, A*β*42 levels decrease as total tau and p-tau levels increase. A*β*40 levels either remain unchanged or increase slightly [66]. CSF A*β* levels stay steady during the first stages of the dementia phase of the disease, but tau levels may show a slight increase [68]. However, in AD cases, reduced A*β*42 along with increased total tau and p-tau levels do not correlate with Mini-Mental Status Examination (MMSE) score. Nonetheless, they suggest a faster decline in cognitive function [69]. The persistent changes in A*β* concentrations in CSF reflect an equilibrium between amyloid synthesis and removal or breakdown [68–70]. AD patients show consistent CSF changes compared to healthy individuals: reduced CSF production, increased cerebral atrophy leading to higher CSF volumes, increased brain-derived protein levels in ventricular CSF versus lumbar CSF, detection of monomeric A*β* species in CSF measures, and A*β* oligomerization contributing to reduced monomeric A*β* levels in AD patients' CSF [70]. Low CSF A*β*42 levels and increased total tau or p-tau levels predict MCI progressing to AD dementia with 83% sensitivity, 72% specificity, 62% positive predictive value, and 88% negative predictive value [71]. Additionally, A*β*-like peptides produced by b- and g-secretase are present in the CSF of AD and MCI patients [72].

Similarly, Alzheimer's disease has been implicated in activating the development of neurofibrillary tangles [73]. Some studies found phosphorylated tau (p-tau) as a more accurate diagnostic marker for AD compared to total tau [73, 74]. These intracellular proteins are known to be released by deteriorating cells, progressing from brain fluid to CSF. In cognitively healthy individuals, CSF A*β*42 correlates with brain decrease, whereas in mild Alzheimer's disease, CSF tau and p-tau are associated with this decrease [49]. Baseline levels of p-tau in CSF serve as predictive indicators for both initial and ongoing hippocampal atrophy in Alzheimer's disease [74]. These findings imply that differences in tau and MRI changes point to neurodegeneration, while changed A*β* levels precede these changes [62]. In recent times, CSF levels of A*β*42, total tau, and p-tau have been linked to clinical outcomes in investigations of healthy older people with amnestic mild cognitive impairment or mild Alzheimer's disease. The total tau to A*β*42 ratio (log scale) predicts the progression from amnestic MCI to Alzheimer's disease [75]. In cases of very mild Alzheimer's disease, lower baseline A*β*42 levels and higher tau or p-tau levels suggest a faster rate of dementia progression [75]. Increased total tau levels correlate with lower scores on MMSE and ADAS-cog assessments [76]. Many CSF proteins have been examined for Alzheimer's disease biomarker development; however, their validation has been confined to small groups of patients. Therefore, before employing these as exploratory markers in trials, they must be confirmed in larger populations by different laboratories [75, 76].  Table 1 presents some of the molecules discussed above, demonstrating differential levels in CSF from Alzheimer's disease patients compared to controls.

### 2.3. Neuroimaging as a Diagnostic Tool

#### 2.3.1. MRI and fMRI

Neuroimaging has become a valuable tool in AD research [60, 77]. Throughout the progression of neuropathological processes, the brain undergoes constant morphological and functional changes [77, 78]. Aging-related changes primarily involve synaptic and neuronal loss, notably more evident in dementia, with variable regional patterns depending on the specific dementia type [77]. However, some anatomical changes are unable to differentiate between these conditions [78]. Neuroimaging techniques are typically categorized as structural or functional based on the primary information they provide [60]. Structural imaging techniques like computed tomography (CT) or magnetic resonance imaging (MRI) are commonly used to examine the different causes of dementia, such as normal pressure hydrocephalus or specific lesions like tumors. They aid in diagnosis by detecting areas of atrophy or vascular abnormalities [43]. CT, on the other hand, provides strong spatial resolution, whereas MRI offers higher contrast resolution. On the other hand, functional imaging techniques like PET or SPECT provide insights into brain anatomy [28], albeit with lower spatial resolution than structural techniques [60, 77, 78]. They do extremely well in measuring brain metabolic factors such as regional cerebral blood flow and glucose metabolism [77, 78]. These functions are often impaired in Alzheimer's disease and other dementias before visible morphological changes, thus, enhancing the usefulness of functional imaging for early diagnosis [32, 71]. In recent years, neuroimaging techniques have been adopted in clinical settings, and ongoing innovations have explored new applications.

MRI generates high-resolution imaging, providing extensive information about brain structure, including the difference between gray and white matter [79]. Individuals with AD typically have parietal lobe and hippocampal atrophy on MRI when compared to controls [79]. Early-onset AD studies using structural MRI identify atrophy in posterior regions like the precuneus, posterior cingulate, amygdala, occipital lobes, corpus callosum, and extensive posterior cortical involvement [80]. Hippocampal and entorhinal cortex atrophy can predict memory decline and is linked to an increased risk of AD development [79, 80]. However, these changes are not limited to AD [81]. Despite its function in clinical diagnosis, structural MRI alone is not definitive due to the substantial overlap between atrophy seen in normal aging and various neurodegenerative conditions, including AD [79, 81]. Nonspecific white matter changes, common in elderly individuals with stroke or mild cognitive impairment, are also prevalent in healthy older adults [79, 81]. Contrarily, various studies demonstrate the diagnostic potential of structural MRI [81, 82]. For instance, individuals with amnestic MCI who later develop AD exhibit more pronounced atrophy in multiple brain regions, including the hippocampus and inferior and middle temporal gyri [82]. Additionally, as AD progresses, the corpus callosum shows anterior atrophy, differentiating it from frontotemporal lobar degeneration (FTLD), where the posterior part of the corpus callosum is primarily affected [83]. The development of high-resolution volumetric MRI and sophisticated automated analysis tools, like voxel-based morphometry, is expected to enhance the detection of subtle abnormal patterns specific to different types of dementia, thereby improving the accuracy of MRI-based diagnoses [79, 81].

Functional MRI (fMRI), on the other hand, examines brain function over time, usually during rest or while performing tasks that activate specific brain regions and networks [84]. A common method, blood oxygen level-dependent fMRI, tracks changes in blood flow linked to neuronal activity, indirectly revealing brain activity. However, its use for diagnosing dementia is limited due to individual variability and reliance on hemodynamics. Nonetheless, fMRI can identify distinct functional deficits linked to different diseases [80, 84]. In Alzheimer's disease, for instance, reduced brain activity in the parietal and hippocampal areas is observed alongside increased activity in other unaffected brain regions compared to healthy individuals [85, 86]. Recent advancements in functional MRI have been instrumental in finding crucial functional networks within the human brain [84]. By exploring cognitive and behavioral functions during the early stages of neurodegenerative disorders, researchers have the potential to identify affected brain networks [87]. This exploration could offer insights into how different neurodegenerative diseases uniquely impact important brain networks [86]. Thus, using fMRI to examine cognitive and behavioral functions in early-stage neurodegeneration provides an avenue to understand the specific network changes in different diseases. This novel approach holds promise for leveraging fMRI as a differential diagnostic tool for various dementia-causing disorders [84, 87].

#### 2.3.2. PET and SPECT

Positron emission tomography (PET) and single photon emission computed tomography (SPECT) imaging using radioactive tracers enable highly sensitive evaluations of physiological functions and protein distribution [88]. These methods are widely used in diagnosing dementia, assessing cognitive decline, and identifying different neurodegenerative diseases. For example, [18F]FDG-PET assesses cerebral glucose metabolism, which indirectly reflects synaptic activity. FDG-PET studies in AD reveal specific patterns of cortical hypometabolism, initially impacting posterior brain regions before progressing to other areas [88]. These distinct metabolic changes seen in AD, differing from both healthy individuals and other forms of dementia, are linked to cognitive decline in individuals with mild cognitive impairment (MCI) [89]. While PET assessment of glucose metabolism boasts high sensitivity (94%) in diagnosing AD, its specificity is lower, ranging from 73% to 78% [88]. Similarly, SPECT, measuring regional blood flow using Tc-hexamethylpropylene amine oxime, shows a comparable level of specificity for diagnosis [90]. PET imaging using low-molecular-weight compounds like Pittsburgh compound B (PIB) displays markedly increased cortical binding in AD patients compared to controls [91]. Despite continuous monitoring, there is no substantial increase in PIB binding observed in AD patients over two years, even with declines in both glucose metabolism and cognitive function [92]. PIB binding patterns vary, with significant binding in CAA, variable binding in LBD, and no binding in FTD [93]. Recent MRI correlations with PIB binding reveal that while higher amyloid deposits do not generally align with more severe gray matter atrophy, there are exceptions in the medial temporal lobes [94]. Another PET ligand, [18F]FDDNP, targets amyloid and tau, offering potential insights into these diseases in healthy individuals [95]. Comparisons between PIB and [18F]FDDNP binding demonstrate a modest correlation, suggesting that they measure related yet distinct disease features, displaying differences in regional binding and findings in MCI [96].

### 2.4. Major Challenges in Blood-Based Biomarkers for Alzheimer's Disease

**Figure 1:**
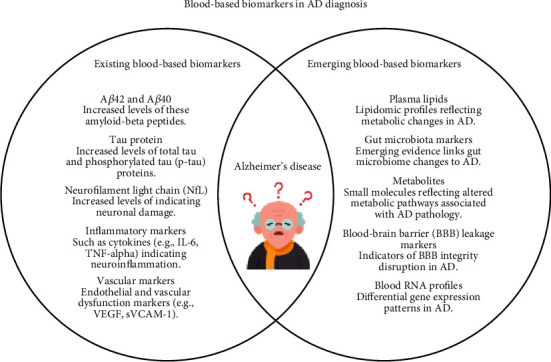
A brief diagram showing existing biomarkers and emerging blood-based biomarkers used in AD diagnosis.

Blood-based biomarkers (see Figure 1), specifically plasma NfL and CSF NfL, have the potential to change the way Alzheimer's disease is diagnosed and treated [79, 97]. This is due to their noninvasive nature, cost-effectiveness, and ability to detect neurodegenerative changes at an early stage. Many studies assess these biomarkers for Alzheimer's treatment implications [98]. Both plasma NfL and CSF NfL show potential as AD diagnostic markers. Increased plasma or serum NfL levels may indicate central nervous system dysfunction, implying their role as markers for AD-related neurodegeneration [99, 100]. Additionally, some studies indicate that plasma NfL is more clinically relevant than CSF NfL, making it suitable for use in the general population [100]. Longitudinal studies also demonstrated that plasma NfL is effective in assessing disease progression and predicting neurodegeneration in older adults with Alzheimer's disease [97, 101]. The ability to monitor disease progression and assess treatment effectiveness is important in managing Alzheimer's disease [98]. The noninvasive collection of blood makes it a feasible method for ongoing monitoring [94, 102]. This is very essential in evaluating the effects of interventions and disease-modifying treatments [102]. Beyond their diagnostic and monitoring roles, blood-based biomarkers could profoundly influence Alzheimer's disease treatment. These biomarkers could contribute to the development and evaluation of disease-modifying medications by aiding the observation of neurodegenerative changes [79]. They might act as objective measures of treatment response and disease progression [98, 103]. Therefore, blood-based biomarkers come with both advantages and challenges [42, 76]. The introduction of new blood-based biomarkers for Alzheimer's disease could optimize clinical trial design [104, 105]. Given Alzheimer's prolonged preclinical phase, these biomarkers may determine trial inclusion criteria, assess treatment effectiveness, and target engagement [95, 99]. Other biomarkers, like those indicating neuroinflammation, are essential for exploring alternative or combination treatments. Blood-based biomarkers are valuable for monitoring treatment effectiveness, analyzing amyloid and tau clearance, and assessing effects (e.g., amyloid-related imaging abnormalities) [36]. Compared to CSF and imaging biomarkers, blood tests offer advantages like noninvasiveness, cost reduction, and lower impact on patients and healthcare systems (e.g., tracer costs and scan duration). However, imaging provides better spatial resolution [35, 87, 97].

Despite their benefits, blood biomarker development faces challenges due to AD's slow progression and uncertainties about blood-brain barrier integrity. Current research seeks blood-based biomarkers for conditions like multiple sclerosis, traumatic brain injury, and stroke. However, in diagnosing Alzheimer's disease, inconsistencies in amyloid imaging and CSF A*β* measurements challenge reliability [106]. This gap in biomarkers versus clinical findings complicates AD research, emphasizing the need for precise disease markers and specific dementia diagnosis to improve treatment options [98, 100]. Using healthy controls for comparison with AD patients may introduce bias due to the multiple medical conditions in AD patients affecting blood-based biomarkers [28, 49]]. Brain pathologies like AD present challenges as they break down slowly within the BBB, impacting the selection of brain-specific protein markers [52, 96]. Complexity results from various factors, such as aging and different dementia risk factors, influencing inflammatory protein levels and complicating the interpretation of plasma profiles [59, 71]. Detecting brain-derived proteins in serum is limited by the BBB's constraints on large protein movement between the brain and peripheral circulation [105]. While some small peptides may cross the BBB, their detection in serum or plasma remains limited. Moreover, although some BBB disruption is seen in AD, current assays lack the sensitivity to measure tau protein in AD patients' blood samples [105, 106]. A*β*, found outside the brain, interacts with multiple blood proteins, demanding careful attention in diagnosis [60, 80]. This interaction poses challenges for blood biomarkers due to low analyte levels, protein binding, and potential brain protein changes, encouraging deeper exploration in diagnostic research. These challenges extend to various target proteins, notably in plasma profiling [32, 76]. Considering the diverse tissue origins of most plasma components, comprehensive screening is essential in Alzheimer's disease and other conditions. This thorough approach facilitates the integration of biomarkers from inflammation, diabetes, and cardiovascular disease, potentially enhancing the effectiveness of AD markers despite inherent uncertainties [104, 107]. The complexity of developing blood-based AD biomarkers stems from the ever-changing blood proteome, affected by various patient factors and external influences [77, 92]. To address this challenge, employing rigorous control measures in blood proteome analysis becomes crucial. One strategy involves leveraging CSF or neuroimaging biomarkers to differentiate AD patients from controls rather than relying solely on clinical criteria [65, 98]. By concentrating on an AD subgroup with distinct CSF A*β*42 levels or positive A*β* neuroimaging, the refinement of blood-based biomarkers becomes possible, reducing the influence of non-AD cases within diagnostic groups. Recent studies have started adopting this approach [99]. For prognosis, strategies might target early disease stages, starting with healthy individuals to detect predictive changes in cognitive decline over time, preferably using brain-specific proteins like tau [52, 105]. Protein fragments from pathological breakdown have shown promise across different diseases [63, 107].

## 3. Associations between CSF and Plasma NfL Concentrations: Differences and Predictive Capabilities

### 3.1. Cross-Sectional Comparisons of Plasma GFAP, T-Tau, p-tau181, p-tau231, and NFL as Predictors of Brain A*β* Status

Neurofilament light emerged as a significant blood-based biomarker indicating neuroaxonal injury, a characteristic prominently observed in Alzheimer's disease [3, 53]. The constant evolution of plasma neurofilament light biomarkers has markedly enhanced the assessment of AD-related pathologies [79, 108]. In parallel, recent advancements in diagnosing Alzheimer's disease have turned towards using several accessible blood tests that measure biomarkers such as amyloid-*β* and tau pathology, both recognized features of the disease, in addition to assessing neurodegeneration [97, 107] In a retrospective study conducted by Benedet et al. [98], an interesting correlation between plasma NfL levels and magnetic resonance imaging measurements of gray and white matter levels in the Alzheimer's Disease Neuroimaging Initiative was explored. The study's findings intriguingly suggested a course in plasma NfL levels: an increase attributed to neuronal injury associated with amyloid during the preclinical stages, followed by a change towards tau-mediated neurodegeneration in symptomatic patients [98, 108] This compelling correlation between plasma NfL and the progression of neuronal injury in different disease stages highlights its potential as a useful biomarker. It not only aids in defining the progression from preclinical to symptomatic phases but also underlines the complex relationship between amyloid and tau pathology in Alzheimer's disease [98, 99]. Moreover, the association between plasma NfL and tau in individuals with cognitive impairment suggests continuous accumulation of tau pathology in this group [109]. This contrasts with the notion that amyloid-beta reaches a plateau during symptomatic Alzheimer's disease [94]. Recent studies highlight the growing importance of plasma NfL levels in predicting progression to all-cause dementia. These findings support the idea that plasma NfL, as a general biomarker of neurodegeneration, can detect changes not specific to Alzheimer's disease (non-AD) [105]. Additionally, Cullen et al.'s study [103] demonstrates that a combination of three plasma biomarkers, including plasma p-tau217, A*β*42/A*β*40 ratio, and plasma NfL, effectively predicts cognitive changes and AD dementia development. Specifically, plasma p-tau217 and the A*β*42/A*β*40 ratio predict both preclinical Alzheimer's cognitive composite (PACC) and AD dementia risk, providing valuable information on cognitive decline [103, 105]. Additionally, plasma NfL significantly indicates PACC and demonstrates greater effectiveness in predicting general cognitive decline and the risk of developing all-cause dementia. Some studies have also demonstrated that progress in using neurofilament light as a biomarker reveals the ability to detect increased NfL levels in blood, suggesting neurodegeneration [109]. This detection can occur in the early stages of autosomal dominant Alzheimer's disease (ADAD) even before the onset of symptoms [101]. There are established associations between cognition and specific blood biomarkers such as glial fibrillary acidic protein (GFAP), phosphorylated tau (p-tau181, p-tau231), and NfL [103, 106]. Notably, increased plasma NfL levels are observed in other neurodegenerative disorders, suggesting its nonspecificity to AD and as a general neurodegeneration biomarker [103]. This is plausible due to the potential impact of correlations between plasma NfL and cerebrospinal fluid NfL on the variability in NfL concentrations observed in AD compared to other conditions [103, 104]. These correlations reflect diverse pathological conditions within AD [108]. However, in mild cognitive impairment, high plasma NfL correlates with low CSF A*β*42 and high CSF total tau [106, 107]. This shows that plasma NfL is a sensitive biomarker for detecting AD-related changes in the early stage [107]. Gerards et al.'s recent study [108] found that plasma NfL is associated with cognitive impairments and MRI characteristics in dementia of Alzheimer's type (DAT) [108, 109]. The study suggests that plasma NfL, along with other biomarkers, can differentiate diagnostic groups, hinting at its potential as a prognostic biomarker in Alzheimer's disease outside of research settings [94, 108, 109]. Consequently, plasma NfL holds promise as a biomarker for detecting neuronal injury in Alzheimer's disease because of its importance in prognosis and monitoring AD progression [94, 105].

### 3.2. Correlation of Plasma NfL with the Four Core CSF Biomarkers

The challenges in reliably diagnosing preclinical and early clinical phases of Alzheimer's disease [110] highlight the urgent need for blood-based biomarkers to aid in the identification of AD-related disorders [111, 112]. Although methods like patient history, neuroimaging, and neuropsychological assessments can identify AD in approximately 80 to 90% of cases, the accuracy of clinical diagnosis can vary. In recent years, cerebrospinal fluid has become a crucial source of biomarkers for neurological diseases. This is attributed to its direct interaction with the brain and its capacity to mirror brain-related metabolic changes [113, 114]. Within this area, the A/T/N system—a biomarker-based biological classification—provides vital insights into Alzheimer's disease. This classification system is based on the evaluation of specific biomarkers: *β*-amyloid, as evaluated by CSF A42 levels using immunoassays or amyloid PET detection, and neurodegeneration, as measured by total tau in CSF using ELISA [112, 115]. This approach provides an effective approach for understanding and diagnosing Alzheimer's disease by using blood-based biomarkers to differentiate between its complex pathophysiological mechanisms [111, 116, 117]. Several studies and meta-analyses consistently demonstrate that individuals with AD had lower CSF A*β*42 concentrations compared to controls [85, 117]. Importantly, evidence supports the predictive value of A*β*42 levels in the progression of individuals with normal cognition and mild cognitive impairment [86, 118]. Lower CSF A*β*42 levels were associated with individuals with mild cognitive impairment who later developed Alzheimer's disease [88], suggesting a potential for faster progression to AD in those individuals [90, 91, 119, 120]. Additionally, hyperphosphorylated tau forms detected in CSF correlate with the observed tangle pathology in the neocortex, indicating that specific forms of CSF p-tau could serve as markers for tangle pathology [121]. Increased levels of tau and p-tau181 have been predictive of progression from mild cognitive impairment to Alzheimer's disease [121], while the tau(s) to A*β*42 ratio consistently predicts cognitive decline [122, 123]. Recent meta-analyses provide evidence supporting the use of baseline levels of CSF A*β*42 and tau as biomarkers for selecting individuals with mild cognitive impairment and AD pathology in clinical trials, significantly reducing sample size and trial costs [119, 121, 124]. While our understanding of Alzheimer's disease has improved, its complex underlying pathophysiology, including amyloid plaque deposition, neuroinflammation, neurofibrillary tangle formation, and neuronal loss, is reflected in the composition of cerebrospinal fluid [124]. However, developing reliable and accurate clinical-grade assays for new CSF biomarkers, implemented on validated and fully automated platforms, is crucial for their routine use in clinical settings to assess individuals identified to have Alzheimer's disease [39, 42, 124].

### 3.3. Plasma and CSF Neurofilament Light in Alzheimer's: Detection, Progression, and Prognosis

Recent studies have shown that both plasma and CSF NfL levels can be predictors of cognitive decline, with some studies suggesting that CSF NfL may be more strongly associated with cognitive decline [109, 125]. When comparing the sensitivity for early detection biomarkers between plasma and CSF NfL, it is important to note that the relationship between NfL levels and cognitive decline may vary based on various factors, including age and disease stage [109, 125–127]. While plasma NfL levels have shown promise as an early detection biomarker due to their significant elevation in AD and mild cognitive impairment patients compared to controls [33], CSF NfL concentrations have demonstrated a stronger association with specific pathological changes, such as amyloid-beta presence during the preclinical stage of sporadic AD [128]. Moreover, CSF NfL levels have been linked to brain atrophy even in individuals without cognitive impairment, suggesting its potential for early detection in asymptomatic individuals [32, 128].

In terms of correlation with disease progression, both plasma and CSF NfL levels have been found to significantly increase during the early stages of sporadic AD and are closely associated with cognitive decline and characteristic structural changes observed in the brain [126, 127]. However, studies focusing on NfL in blood have not consistently demonstrated such changes during this early stage of the disease [42, 76]. These findings suggest that CSF NfL might be a more reliable biomarker for detecting neurodegenerative processes in preclinical sporadic AD compared to plasma NfL [76]. The study conducted by Pereira et al. [32] using the Alzheimer's Disease Neuroimaging Initiative cohort revealed a novel finding: greater concentrations of CSF NfL were connected to brain atrophy, even in patients without cognitive impairment but with abnormal CSF A42 levels. Remarkably, this study also observed increased plasma NfL levels associated with brain atrophy, specifically in symptomatic individuals [32]. The strong association between NfL levels in both CSF and blood indicates their relevance to the preclinical stages of various neurodegenerative diseases, providing valuable insights into early disease progression [125–127, 129, 130]. Additionally, plasma NfL has shown associations with brain imaging measurements, risk factors for AD, and cognitive performance [131, 132]. All these findings suggest that plasma levels of A*β*42/A*β*40, p-tau isoforms, and NfL reflect the underlying pathology of AD and have the potential to serve as valuable prognostic biomarkers for monitoring disease progression. Few studies have directly compared the predictive abilities of plasma and cerebrospinal fluid biomarkers for cognitive decline within the same group of individuals. These studies have generally indicated similarities between CSF and plasma in terms of their predictive capabilities. For instance, Li et al. [131] conducted a comparison of CSF and plasma levels of NfL and observed similar effect sizes in both analyses. However, only plasma NfL demonstrated a significant association with cognition, while CSF NfL did not show a significant association [131]. In contrast, Martinez et al. [132] reported that plasma and CSF markers of amyloid performed similarly, but only CSF markers of total tau were predictive of cognitive change, whereas plasma total tau was not [132]. Additionally, Dong et al. [133] found that cerebrospinal fluid measures outperformed plasma measures in predicting a longitudinal decline, even though the analysis employed a less effective performance metric for plasma A*β*42/A*β*40.

Regarding prognostic value, both plasma and CSF biomarkers have shown predictive capabilities for cognitive decline. While studies have generally indicated similarities between CSF and plasma NfL in terms of their predictive capabilities [133], some differences have been observed. For example, plasma NfL has shown a significant association with cognition in certain studies, suggesting its potential as a prognostic marker for cognitive decline [134, 135]. However, CSF measures of total tau have been reported to be predictive of cognitive change, whereas plasma total tau was not, indicating potential differences in their prognostic value [135]. Despite these discrepancies, CSF measures have been reported to outperform plasma measures in predicting longitudinal decline, emphasizing the importance of considering both biomarkers in prognostic assessments [103, 105, 125, 136, 137]. Thus, while both plasma and CSF NfL levels hold promise as biomarkers for early detection and monitoring of AD progression, CSF NfL may offer advantages in terms of reliability for detecting neurodegenerative processes in preclinical AD [138]. However, both biomarkers demonstrate strong associations with cognitive decline and disease progression, highlighting their potential utility in prognostic assessment [139, 140].  Table 2 presents blood-based biomarkers for Alzheimer's disease, detailing their respective biomarker, blood/fluid matrix, observations in AD, and interpretations/applications.

## 4. Conclusion and Future Perspectives

Blood-based biomarkers have shown promise in detecting AD-related pathologies and monitoring disease progression. Despite the use of other biomarkers and imaging techniques in AD diagnosis, their limitations in accessibility, invasiveness, and potential for early detection highlight the significance of blood-based biomarkers. The review found that both plasma and CSF NfL levels predict cognitive decline, although some studies support CSF NfL. Increased CSF NfL levels were identified in cases of brain atrophy, even among cognitively healthy individuals with abnormal CSF A*β*42 levels. Similarly, elevated plasma NfL levels were associated with brain atrophy in symptomatic individuals. Also, NfL concentration in CSF indicates neuroaxonal injury across neurological conditions. These findings suggest that the concentration of NfL in CSF could serve as a valuable biomarker for evaluating axonal injury and loss in the preclinical stages of sporadic AD. Meanwhile, the concentration of NfL in plasma might reliably indicate similar pathological processes, albeit at a later stage, specifically during the early symptomatic phases of the disease.

Therefore, the integration of blood-based biomarkers for Alzheimer's disease presents a promising avenue for advancing diagnostic tools and management. These biomarkers offer the potential for less invasive and more accessible diagnostic tools, which could redefine AD diagnosis and management. To achieve this, further research should focus on strong validation studies to establish the reliability, accuracy, and reproducibility of these blood-based indicators in diverse clinical settings. Additionally, exploration into expanding the range of blood-based biomarkers beyond existing ones and investigating novel markers with enhanced specificity and sensitivity is crucial. The research also indicates that both plasma and CSF NfL levels can predict cognitive decline, with some studies suggesting that CSF NfL may be more strongly associated with cognitive decline. However, the relationship between NfL levels and cognitive decline may vary based on age and other factors. Further research is needed to fully understand the potential of plasma and CSF NfL as predictors of cognitive decline. Additionally, plasma NfL serves as a nonspecific marker for neurodegeneration, potentially indicating increased levels in various neurodegenerative disorders. Thus, additional research is essential to determine the specificity of these biomarkers for Alzheimer's disease and their ability to differentiate it from other conditions.

## Figures and Tables

**Table 1:** Molecules that showed differential levels in CSF from Alzheimer's disease patients as compared to controls.

Protein	Up/downregulated
Albumin	↑
Amyloid *β* A4 protein	↑
Apolipoprotein AI	↑
Apolipoprotein AII	↑
Apolipoprotein E	↑
BACE 1	↑
24S-Hydroxycholesterol	↑
C3a	↑
C4a	↑
Cystatin C	↑
Cystatin C, 8 amino acid N-terminal truncation	↑
Immunoglobulin heavy chain	↓
Leucine-rich repeat-containing protein 4B	↑
Leucine-rich repeat-containing protein 4B	↓
N-acetyllactosamine	↓
Neuronal pentraxin-1	↑
Prostaglandin-H2 D-isomerase	↑ ↓
Retinol-binding protein	↑
Thioredoxin	↑
Transthyretin	↓ ↑ ↓
VGF	↑
a-1-Antitrypsin	↑
*α*-1*β* glycoprotein	↑
*α*-2HS glycoprotein	↓
*β* fibrinogen	↑
*β*-2-Microglobulin	↓

**Table 2:** Blood-based biomarkers for Alzheimer's disease.

Biomarker	Blood/fluid matrix	Observation in AD	Interpretation/application
NFL	Blood (plasma or serum)	Increased levels were observed in Alzheimer's disease, familial Alzheimer's disease, and the early stages of Alzheimer's disease.	Increased plasma NFL serves as a broad indicator of neurodegeneration, not exclusively linked to Alzheimer's disease. It could potentially serve as a screening tool for detecting general neurodegeneration.

A*β*42	CSF	Reduced A*β*42 in Alzheimer's disease and its early stages (with a sensitivity of over 90%).	Indicates the presence of A*β* accumulation in the brain. Established as a diagnostic marker with two fully verified mass spectrometry reference measurement procedures (RMP) approved.
	Blood (plasma)	Immunoaffinity-based mass spectrometry (IP-MS) indicates lower levels of plasma A*β*42 in Alzheimer's disease. Plasma A*β*42 concentrations exhibit a mild to moderate agreement with amyloid PET.	Indicates amyloid deposition in the brain but is impacted by peripheral expression. A potential tool for screening purposes.

p-tau	CSF	Increased p-tau is observed in Alzheimer's disease and its early stages (sensitivity > 90%).	Increased p-tau levels indicate tau's phosphorylation status, likely reflecting tau pathology in Alzheimer's disease. p-tau is more AD-specific compared to T-tau and serves as a diagnostic biomarker.
	Blood (plasma)	Higher p-tau levels appear specific to AD cases with A*β* positivity. There is an association between amyloid PET and tau PET (assessed through MSD assay).	Potential biomarker for diagnosing and screening purposes.

A*β*42/A*β*40	CSF	Reduced A*β*42/A*β*40 ratio is observed in Alzheimer's disease and its early stages. Higher accuracy (sensitivity and specificity) compared to A*β*42 alone	The A*β*42/A*β*40 ratio is aimed at adjusting for differences in “total” A*β* production among individuals. Biomarkers used for diagnosis.
	Blood (plasma)	Simoa and IP-MS show reduced plasma A*β*42/40 in AD and prodromal AD. Plasma A*β*42/40 ratio moderately aligns with amyloid PET results.	The A*β*42/A*β*40 ratio might indicate cerebral amyloidosis-related mechanisms. A potential tool for screening.

Neurogranin	CSF	Higher neurogranin was observed in Alzheimer's and early stages of the disease.	Indicates synaptic dysfunction or degeneration. Biomarkers used for diagnosis.

T-tau	CSF	Increased T-tau levels are present in Alzheimer's disease and its early stages (with sensitivity exceeding 90%)	Increased T-tau indicates the severity of neurodegeneration. Biomarkers used for diagnosis.
	Blood (plasma)	Slight to moderate increases were observed in Alzheimer's disease and its early stages.	Affected by external or peripheral expression Not likely to serve as a biomarker in Alzheimer's disease
